# Mechanical Properties of the New Generation RACE EVO and R-Motion Nickel–Titanium Instruments

**DOI:** 10.3390/ma15093330

**Published:** 2022-05-06

**Authors:** Fatima Betul Basturk, Taha Özyürek, Gülşah Uslu, Mustafa Gündoğar

**Affiliations:** 1 Department of Endodontics, Istanbul Gelişim University, Istanbul 34310, Turkey; 2 Department of Endodontics, Bahçeşehir University, Istanbul 34734, Turkey; taha.ozyurek@dent.bau.edu.tr; 3 Department of Endodontics, Çanakkale Onsekiz Mart University, Çanakkale 17100, Turkey; gulsahuslu@comu.edu.tr; 4 Department of Endodontics, Istanbul Medipol University, Istanbul 34083, Turkey; mustafagundogar@msn.com

**Keywords:** bending resistance, dynamic cyclic fatigue resistance, differential scanning calorimetry, reciprocation, torsion

## Abstract

This study aimed to evaluate and compare the dynamic cyclic fatigue, torsional and bending resistance of two novel RACE EVO (FKG Dentaire SA, La Chaux de Fonds, Switzerland) and R-Motion (FKG) nickel–titanium instruments with traditional RaCe (FKG) instruments. RACE EVO, R-Motion and RaCe instruments with a size of 25 and taper of 0.06 were used. A dynamic cyclic fatigue test was used to assess the time to fracture. The fractured surfaces were further analyzed using scanning electron microscopy at ×350 and ×3000 magnifications. A torsional resistance test was performed to measure the maximum torsional strength and angle of rotation. Phase transformations with temperature were evaluated using differential scanning calorimetry. The results were statistically analyzed with a Kruskal–Wallis test at a 5% significance level. R-Motion had the highest time to fracture and the lowest torsional and bending resistance, whereas RaCe had the lowest time to fracture and the highest torsional and bending resistance (*p* < 0.05). In relation to the angle of rotation, RACE EVO instruments had the highest deformation capacity followed by R-Motion and RaCe instruments (*p* < 0.05). The greater cyclic fatigue resistance and lower torsional and bending resistance results indicate that the novel R-Motion and RACE EVO instruments are less rigid and more flexible than RaCe instruments.

## 1. Introduction

In a survey analyzing the need for endodontic referral amongst general practitioners, the major reason for this type of referral was the obstruction of the root canals, mainly by a broken instrument [1]. The most common complication during root canal preparation is intracanal instrument fracture, which is attributed to torsional or cyclic fatigue or a combination of both [2].

Torsional fracture occurs when any part of the instrument is locked in the canal, while the other part of the instrument continues to rotate [3,4]. Cyclic fatigue occurs when the instrument is subjected to repetitive compression and tension cycles in a curved root canal [5].

Various attempts, such as heat treatment and instrument kinematics, have been made to enhance the mechanical properties and flexibility of NiTi (nickel–titanium) instruments [6]. Heat treatments modify the phase-transformation temperature of NiTi instruments to control the instrument’s phase composition at its operating temperature and improve its mechanical and metallurgical properties [7]. Reciprocation motion is also considered an effective way to extend the cyclic fatigue life of NiTi instruments [8].

An ideal endodontic instrument should be flexible with a high torsional resistance and angular deflection under torsional forces. However, these characteristics are not always achievable together. FKG manufactured heat-treated RACE EVO (FKG Dentaire SA, La Chaux de Fonds, Switzerland) for minimally invasive root canal preparation and reciprocating R-Motion (FKG Dentaire) instruments. RACE EVO instruments retain the same design features as RaCe. New electropolished files have a triangular cross sectional design, alternating cutting edges and a rounded tip like their first version. The manufacturer of these RaCe instruments claims that they have an increased cyclic fatigue resistance and flexibility [9,10,11]. R-Motion instruments are reciprocating instruments with a rounded triangular cross-section with sharp cutting edges and thinner core size to reduce the stress on dentine during root canal treatment [10]. Both R-Motion and RACE EVO instruments underwent heat treatment processes and are recognizable from their blue color [9,10].

Yet, there is a lack of information about the mechanical properties of these novel instruments. Therefore, the objective of the study was to evaluate and compare the dynamic cyclic fatigue and torsional resistance, angle of rotation, bending resistance and phase transformations of novel RACE EVO and R-Motion instruments with the traditional RaCe instruments to establish baseline data for further studies.

The null hypothesis was that there would be no difference between the tested instruments regarding mechanical properties.

## 2. Materials and Methods

The sample size was detected using G*Power 3.1 software for Windows (Henrick Heine-Universitat, Düsseldorf, Germany). Based on a previous study [12], a power calculation with the F test family, 0.62 effect size, 0.05 alpha type error and 0.95 power indicated that the minimum total sample size should be 45 per test. Thus, 45 instruments were allocated from each group for dynamic cyclic, torsional, and bending resistance tests (*n* = 15 per group).

RaCe, RACE EVO and R-Motion instruments with a size of 25 and taper of 0.06 were inspected for any defects or deformities under ×20 magnification (OMG 2350, Zumax, Suzhou, China), and none of the instruments were discarded.

### 2.1. Dynamic Cyclic Fatigue Resistance

**Figure 1:**
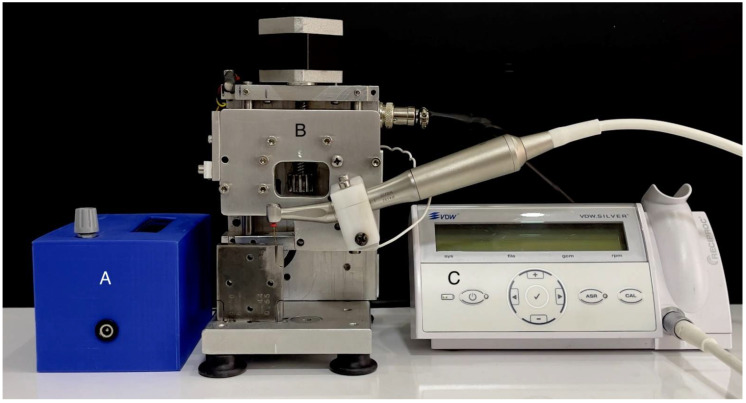
Dynamic cyclic fatigue test setting: (**A**) control panel, (**B**) dynamic device and artificial canal, and (**C**) VDW Reciproc Silver.

Cyclic fatigue resistance of the files was tested using an artificial canal made of stainless steel with an inner diameter of 1.5 mm, 5 mm radius of curvature, and 60° angle of curvature. The cyclic fatigue device was set at 3 mm/s with a 5 mm front–rear axial motion at 36 °C in distilled water bath, in order to simulate clinical conditions. The VDW Reciproc Silver (VDW, Munich, Germany) endodontic motor was connected to the test device and run at 1000 rpm according to the manufacturer’s recommendations until the files fractured (Figure 1). Time to fracture (TTF) was recorded in seconds for each type of file via a digital chronometer.

Two files from each brand were evaluated and photographed under a scanning electron microscope (JSM-7001F; JEOL, Tokyo, Japan) to determine the fracture type.

### 2.2. Bending Resistance

The X-Smart endodontic motor was vertically adapted in a universal testing machine. The file shank was inserted into a standardized groove in a heat-resistant container filled with distilled water at 36 °C. A 20 N load cell attached to the machine head was used. The crosshead speed was set at 2 mm/min. Load was applied at a point 3 mm from the tip of the tested instrument using a stainless-steel blade, which was connected to the universal testing machine (Instron Model 5982, Canton, MA, USA). The force in “gf” unit to bend the file was recorded at the time of 45° displacement.

### 2.3. Torsional Resistance

**Figure 2:**
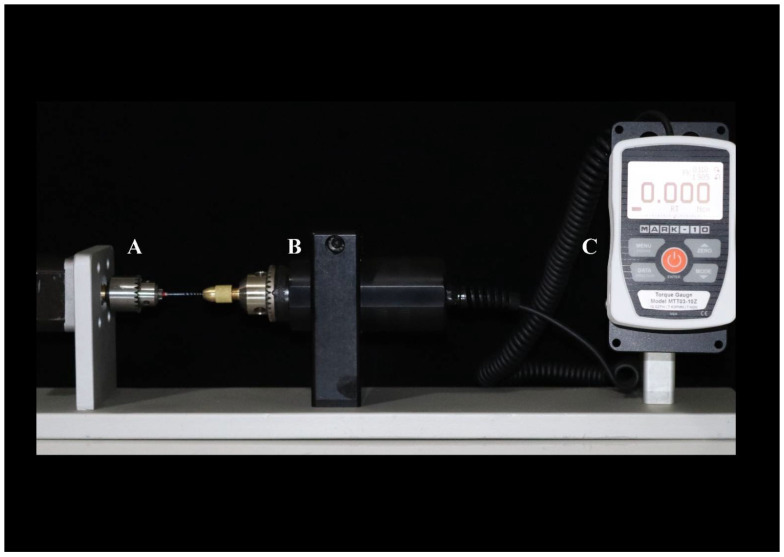
Torsional strength test setting: (**A**) file clip, (**B**) torsion device, and (**C**) control panel.

The torsion test was performed by assessing the torque values by measuring the force applied on a load cell by a lever arm connected to the torsion axis. Measurement and control of the rotation angle was performed by a resistive angular transducer connected to a process controller. The instruments were clamped 3 mm from their tips to the set up (Figure 2). The test was conducted in a clockwise rotation with the speed of 2 rpm, until instrument fracture. The maximum torque and angular deflection to failure were recorded.

### 2.4. Differential Scanning Calorimetry

The fragments cut in 4–5 mm in length were prepared from three representative instruments from each brand. The phase transformation behavior of the tested instruments was analyzed by DSC (Perkin Elmer DSC Pressure; Waltham, MA, USA). For DSC analysis, each instrument was heated from 25 to 100 °C and then cooled to −80 °C at a rate of −10 °C/min, which was immediately followed by a heating cycle at 10 °C/min up to 100 °C. These cycles were repeated three times for each instrument. The heating and cooling curves were recorded and analyzed to detect phase-transformation temperatures.

### 2.5. Scanning Electron Microscopy

Two instruments from each group were randomly selected and examined by a scanning electron microscope (SEM; JEOL, JSM-7001F; Tokyo, Japan) to assess the topographic features of the fractured surface of the instruments. The photomicrographs were taken between ×350 and ×3000 magnification.

### 2.6. Statistical Analysis

The data were analyzed using the SPSS 21.0 (IBM-SPSS Inc., Chicago, IL, USA) software. Kruskal–Wallis test was applied to the data (for TTF, FL, torsion, angle of rotation, and bending) that did not meet the normality assumption as a result of Shapiro–Wilk test, and then a Dunn–Bonferroni test was used for pairwise comparisons. All analyses were performed at the 5% significance level.

Since the data did not show normal distribution, the relationship between TTF and the other recorded values (torsion, bending, and angle of rotation) of the tested instruments was examined by Spearman correlation analysis.

## 3. Results

The median, minimum (min) and maximum (max) values for time to fracture in seconds (TTF) and fractured fragment length in mm (FL) of RaCe, RACE EVO and R-Motion NiTi instruments were presented in Table 1.

R-Motion demonstrated the highest TTF, followed by RACE EVO, and RaCe instruments (*p* < 0.05). There was no significant difference in the FL between the tested files (Table 1). RaCe had the highest torsional strength and lowest angular deflection compared to RACE EVO and R-Motion NiTi instruments (*p* < 0.05) as shown in Table 2.

**Figure 3:**
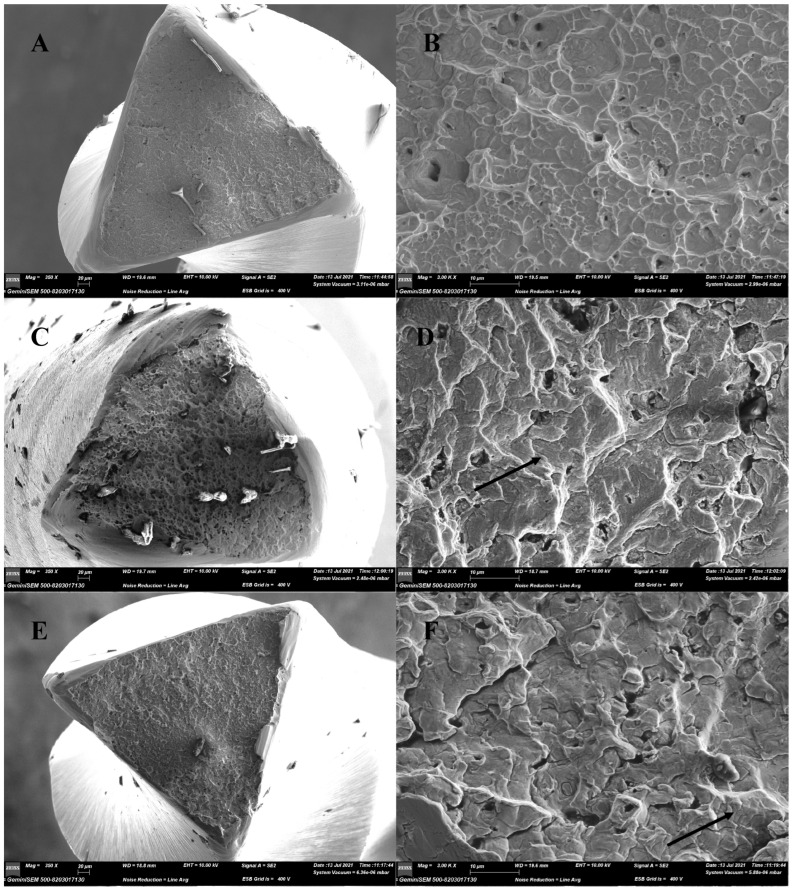
Scanning electron microscopic (SEM) images at lower and higher magnifications for the RACE EVO (**A**,**B**), R-Motion (**C**,**D**) and RaCe (**E**,**F**) instruments’ fractured surfaces after cyclic fatigue test. At a low magnification (**A**,**C**,**E**), the surfaces appeared rough, and at a higher magnification (**B**) microscopic dimples characteristic of cyclic fatigue were observed. When the edges were carefully examined at high magnification (**D**,**F**), fatigue lines due to cyclic fatigue were seen in the areas indicated by the arrows.

A fractographic analysis of the instruments subjected to cyclic fatigue resistance tests showed typical features of cyclic fatigue failure: fatigue striations at margins and wide fibrous area in the center of the instruments under high magnification (Figure 3).

**Figure 4:**
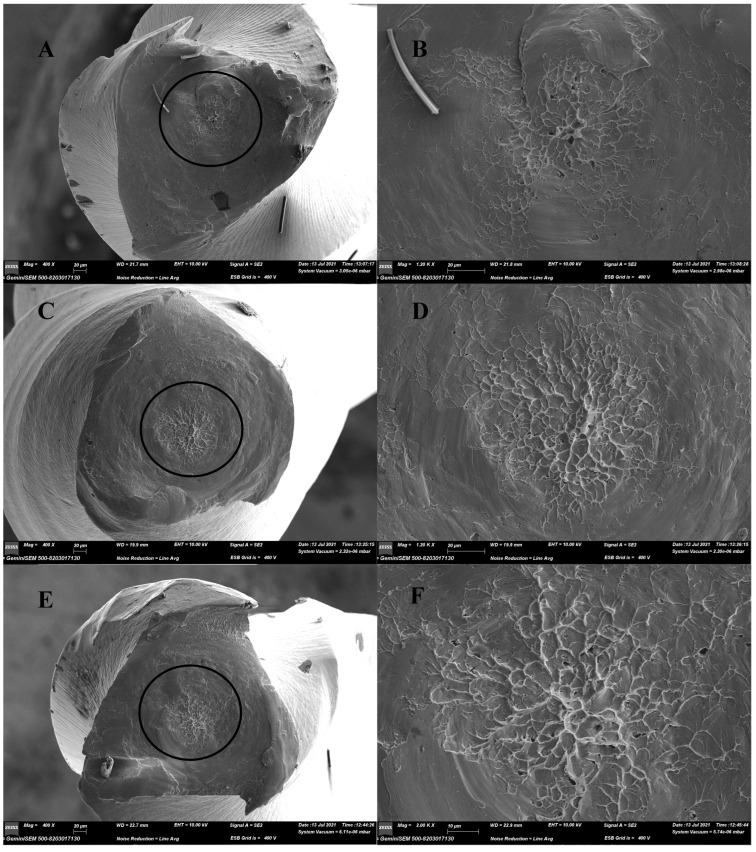
Scanning electron microscopic (SEM) images at lower and higher magnifications for the RACE EVO (**A**,**B**), R-Motion (**C**,**D**) and RaCe (**E**,**F**) instruments’ fractured surfaces after torsion tests. Relatively smooth surfaces compared to cyclic fatigue and concentric abrasion patterns were observed at low magnification (**A**,**C**,**E**). Higher magnification of the central areas indicated by circles at low magnification revealed areas with dimpled surfaces, which are characteristic of torsional fatigue (**B**,**D**,**F**).

The instruments subjected to torsional tests revealed a burnished fracture surface with concentric abrasion marks and a central area with a dimpled appearance where the instrument suddenly ruptured (Figure 4).

**Figure 5:**
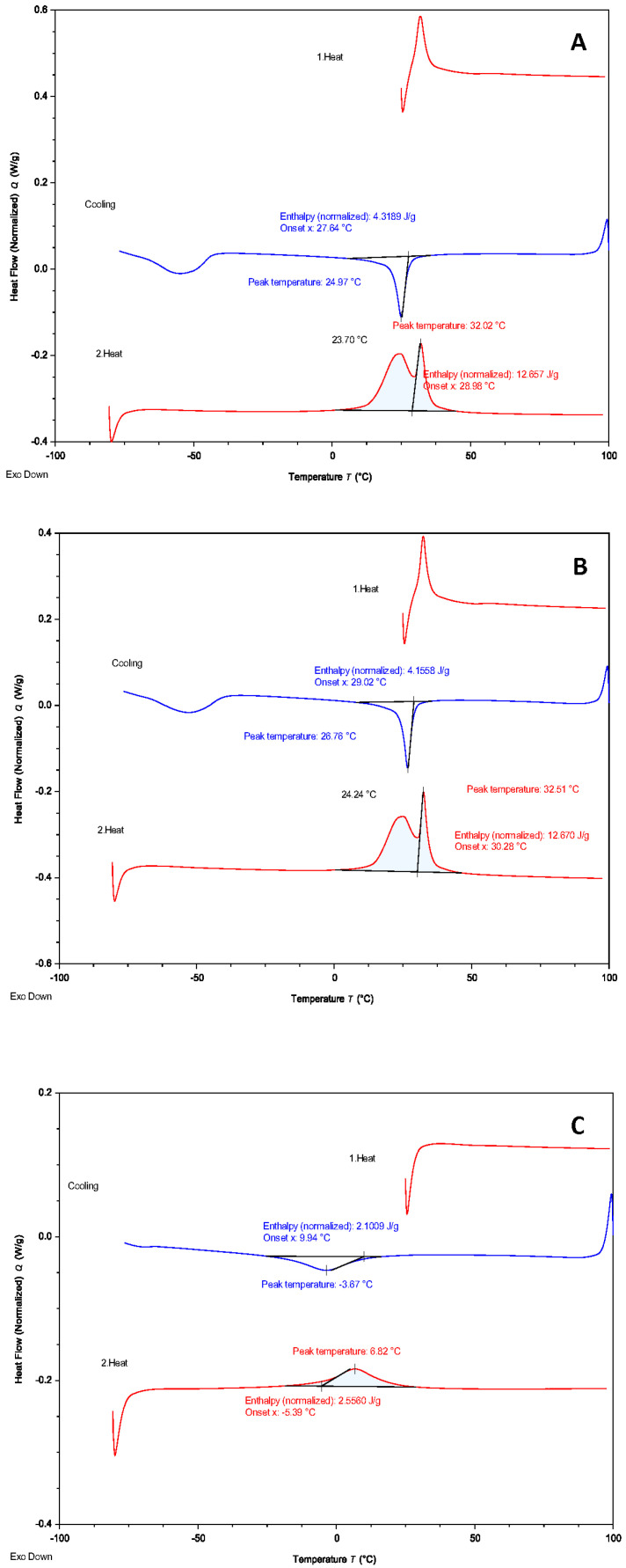
Differential scanning calorimetry (DSC) heating (red lines) and cooling (blue lines) curves of the RACE EVO (**A**), R-Motion (**B**) and RaCe (**C**) instruments.

DSC curves for RACE EVO and R-Motion showed a single peak on the cooling curve and double peak on the heating curve, whereas the DSC curve for RaCe showed a single peak on the cooling and the heating curves (Figure 5).

The austenite start temperatures of RACE EVO, R-Motion and RaCe were 23.7 °C, 24.24 °C, and −5.39 °C, respectively. The austenite finish temperatures (Af temperatures) of tested instruments were determined by locating the intersection of the tangent of the steepest endothermic slope at the austenitic end with the baseline extension of the heating curve [7]. The Af temperatures of RACE EVO, R-Motion and RaCe were 32.02, 32.52 and 6.82 °C, respectively. There was a moderate negative correlation between TTF and bending, torsional resistance, and the angle of rotation when a Spearman correlation analysis was conducted (Table 3).

## 4. Discussion

The mechanical properties of the instruments determine their behavior in the clinic and clinicians’ selection of the file according to the case situation [13]. In the present study, new generation RACE EVO and R-Motion instruments were mechanically analyzed and compared to traditional RaCe instruments. The results show that RACE EVO and R-Motion instruments had the highest cyclic fatigue resistance and angle of rotation, whereas RaCe had the greatest torsional and bending resistance, indicating that the novel instruments were more flexible and less resistant to torsion than their predecessors. Therefore, the null hypothesis was rejected.

Instrument tip size, taper, kinematics and manufacturing methods may affect the torsional and cyclic fatigue resistance of endodontic instruments [5,14,15]. This is the reason why we compared instruments with the same size and taper (size 25, taper 0.06). However, their kinematics and manufacturing methods vary. Additionally, environmental temperature can affect the heat-treated NiTi files’ behavior in root canals at body temperature, and in the cyclic fatigue tests, it is highly recommended that tests are conducted at body temperature [16,17,18]. So, we conducted cyclic fatigue and bending resistance tests in a 36 °C distilled water bath to emulate clinical conditions, despite the fact that previous studies did not [19,20]. It was claimed that the environmental temperature could not affect the torsional strength of the instruments, so this test was not conducted under body temperature [21].

The mechanical tests do not simulate the clinical use of the instruments. However, they provide standardized conditions for all groups [5]. The dynamic test modality features front–rear axial movement and simulates the clinical situation [6,22,23,24]. The dynamic cyclic fatigue resistance is significantly higher than the static resistance in continuously rotating instruments, but not in reciprocating instruments [6]. Fractured fragment length of the files were similar among the groups, which shows that the instruments were similarly well positioned in the curved root canals and were tested at the curved part of the stainless-steel canal in a similar trajectory [25].

Cyclic fatigue resistance is one of the most studied properties of NiTi endodontic instruments [5,26,27]. We found that the cyclic fatigue resistance values of new of RACE EVO and R-Motion instruments were statistically higher than those of traditional RaCe instruments. R-Motion had the highest cyclic fatigue resistance compared to the other instruments. This could be attributed to the kinematics of the R-Motion [28]. Most studies report that, compared to continuous rotation, reciprocating motion increases the fatigue resistance of endodontic instruments [29,30].

A more flexible instrument would experience less stress, allowing for a longer fatigue life span [31]. The manufacturer claimed that a proprietary thermal treatment during the manufacturing process increased the flexibility of RACE EVO [9] and R-Motion instruments [10]. Thermal treatment was applied to the NiTi instruments to improve flexibility affect the crystallographic state, which, consequently, altered their mechanical properties [7,32,33]. A DSC test was conducted to explain the mechanical features of the tested NiTi files [16]. The DSC results revealed that the Af temperatures of both RACE EVO and R-Motion were close to but lower than body temperature, and these files contained more martensite than the RaCe instruments at ambient temperature. It was reported that NiTi alloys in the martensitic crystallographic state were more flexible and more resistant to cyclic fatigue than in the austenitic state [7]. In the present study, both R-Motion and RACE EVO instruments had a higher martensite phase compared to RaCe at ambient temperature.

Bending resistance tests can provide an important clue as to whether an instrument can follow the original canal path in curved root canals [34]. According to the bending resistance test, RACE EVO and R-Motion instruments had a greater flexibility compared with the traditional RaCe instruments. RACE EVO and R-Motion also showed a high angle of rotation before fracture and lower torsional resistance, compared to RaCe instruments. The enhanced flexibility of RACE EVO and R-Motion instruments would be beneficial for tracking the original root canals with less transportation and ledges and with less file fracture, but a higher torsional strength indicates that the RaCe instruments can better resist twisting in calcified or constricted root canals [22].

Different cross-sectional designs, tip and taper dimensions, pitch lengths, and operative motions could affect the resistance to both the flexural and torsional stresses [16,24,35]. However, their cyclic fatigue and torsional resistance values vary. When two instruments have equal cross-sectional areas and mass values, the instrument that is spread less from the pivot center has a lower polar moment of inertia and a lower torsional resistance [36]. The polar moment of inertia is considered a significant factor in between instruments that have similar dimensions and mass volumes, yet differ in their cross-sectional shapes. The continuous rotating instruments used in this study were chosen for their similar cross-sectional designs, and tip and taper dimensions. For files with same geometry and design, thermal treatment enhances the cyclic fatigue lifespan, while at the same time, reducing the torsional resistance of a file [16,35,37]. Thermal treatment alters the martensitic transformation and dissipation of energy required for crack formation and/or propagation during cyclic fatigue testing [37,38,39]. The results of the present study are in accordance with the results from the literature, which showed that thermal treatment can improve flexibility but not torsional strength [40,41]. Table 3 shows the negative correlation between them.

The purpose of a fractographic analysis with SEM images taken after fatigue tests is to confirm that the files were broken due to torsional or cyclic fatigue [4]. Striation clusters related to crack progression were seen on the fracture surfaces, which were observed due to cyclic fatigue. Each line represented the tensile stress that the file was subjected to during rotation. Fractures from the periphery towards the center were caused by the cyclic fatigue progress. Eventually, ductile fracture was observed in the center of the fracture surface [27,35]. After torsional fracture, the file surfaces represented typical shear-induced fracture characteristics, with concentric abrasion marks at the center of rotation and fibrous microscopic pits [30]. The images obtained after the fracture of the RACE EVO and R-Motion files showed typical features of torsional and cyclic fatigue.

One of the main limitations of this study is that the mechanical tests do not necessarily reflect the behavior of the instruments under clinical conditions. Design and manufacturing methods can give insights into the performance of the instruments in the root canals. Intracanal instrument fracture occurs by a combination of cyclic fatigue and torsional stress [14]; the cyclic fatigue resistance of an instrument cannot yet be simultaneously measured with torsional stress. All tests were conducted separately and each of them showed a different mechanical property performed under well-described conditions. However, all of these factors can contribute to fracture in different ways in a non-standardized root canal environment. Nevertheless, considering the characteristics, curvature or narrowness of the root canals, these tests are important in terms of showing the performance of the files under different conditions. Further clinical studies are needed to determine the relationship between the present data and the efficacy of RACE EVO and R-Motion instruments in vivo.

The results of a greater cyclic fatigue resistance and higher angle of rotation, as well as lower torsional and bending resistance, indicate that the novel R-Motion and RACE EVO instruments are more flexible and less resistant to fracture than the RaCe instruments. R-Motion and RACE EVO instruments have higher austenite finish temperature than the RaCe instruments.

## Figures and Tables

**Table 1:** Median, minimum (min) and maximum (max) values for time to fracture as second (TTF) and fractured fragment length as mm (FL) of RaCe, RACE EVO and R-Motion NiTi files.

Groups	TTF Median (Min–Max)	FL Median (Min–Max)
RaCe	583.91 (497.86–789.78) ^c^	5.63 (5.04–5.85) ^a^
RACE EVO	706.53 (598.42–789.78) ^b^	5.69 (5.09–5.91) ^a^
R-Motion	1579.16 (1365.39–1965.12) ^a^	5.59 (5.42–5.80) ^a^

^a,b,c^ Different superscript letters in the same column indicate statistically significant differences among groups (*p* < 0.05).

**Table 2:** Median, minimum (min) and maximum (max) values for torque (N·cm), angle of rotation (°), and bending resistance (N·cm) of RaCe, RACE EVO and R-Motion NiTi files.

Groups	Torsion (N·cm)	Angle of Rotation (°)	Bending (N·cm)
RaCe	1.89 (1.83–2.28) ^a^	552.31 (498.99–599.38) ^b^	2.06 (1.77–2.14) ^a^
RACE EVO	1.26 (1.22–1.52) ^b^	670.56 (602.36–985.64) ^a^	1.37 (1.18–1.43) ^b^
R-Motion	1.27 (1.22–1.58) ^b^	642.23 (572.24–936.35) ^a^	1.29 (1.10–1.1.58) ^b^

^a,b^ Different superscript letters in the same column indicate statistically significant differences among groups (*p* < 0.05).

**Table 3:** Correlation of the time to fracture (TTF) with bending, torsional resistance and angle of rotation of the tested NiTi files.

	Bending Resistance (N·cm)	Torsional Resistance (N·cm)	Angle of Rotation (Degree)
Time to fracture (sec)	r = −0.68 *; *p* < 0.001	r = −0.574 *; *p* < 0.001	r = −0.427 *; *p* = 0.01

r = Spearman correlation coefficient. * Correlation is significant at the 0.01 level (2-tailed).

## Data Availability

The data that support the findings of this study are available from the corresponding author, [FBB], upon reasonable request.
